# Increased Amygdala and Visual Cortex Activity and Functional Connectivity towards Stimulus Novelty Is Associated with State Anxiety

**DOI:** 10.1371/journal.pone.0096146

**Published:** 2014-04-22

**Authors:** Olga T. Ousdal, Ole A. Andreassen, Andres Server, Jimmy Jensen

**Affiliations:** 1 NORMENT, KG Jebsen Centre for Psychosis Research, Division of Mental Health and Addiction, Oslo University Hospital and Institute of Clinical Medicine, University of Oslo, Oslo, Norway; 2 Department of Radiology, Haukeland University Hospital, Bergen, Norway; 3 Department of Neuroradiology, Oslo University Hospital, Oslo, Norway; 4 Centre for Psychology, Kristianstad University, Kristianstad, Sweden; Institute of Psychology, Chinese Academy of Sciences, China

## Abstract

Novel stimuli often require a rapid reallocation of sensory processing resources to determine the significance of the event, and the appropriate behavioral response. Both the amygdala and the visual cortex are central elements of the neural circuitry responding to novelty, demonstrating increased activity to new as compared to highly familiarized stimuli. Further, these brain areas are intimately connected, and thus the amygdala may be a key region for directing sensory processing resources to novel events. Although knowledge regarding the neurocircuit of novelty detection is gradually increasing, we still lack a basic understanding of the conditions that are necessary and sufficient for novelty-specific responses in human amygdala and the visual cortices, and if these brain areas interact during detection of novelty. In the present study, we investigated the response of amygdala and the visual cortex to novelty, by comparing functional MRI activity between 1^st^ and 2^nd^ time presentation of a series of emotional faces in an event-related task. We observed a significant decrease in amygdala and visual cortex activity already after a single stimulus exposure. Interestingly, this decrease in responsiveness was less for subjects with a high score on state anxiety. Further, novel faces stimuli were associated with a relative increase in the functional coupling between the amygdala and the inferior occipital gyrus (BA 18). Thus, we suggest that amygdala is involved in fast sensory boosting that may be important for attention reallocation to novel events, and that the strength of this response depends on individual state anxiety.

## Introduction

Our visual system typically receives several competing stimuli simultaneously. Still, awareness and elaboration are focused on a few stimuli, illustrating at least in part the biasing effects of top-down modulatory mechanisms on visual processing [1], [2]. Novel stimuli boost visual cortex activity, in which both the sensory features of a novel stimulus and gating by other brain areas probably determine the visual responses [3], [4]. The amygdala represents a candidate region for such top-down gating of the visual cortex for novel events, due to its discrimination of novelty [5], [6], [7], [8], intimate connectivity with the ventral visual stream [9], [10] and modulation of visual cortex responses to emotional events [11], [12].

The amygdala shows reliable responses to both auditory [13] and visually [5], [6], [7], [8] presented novel stimuli. This response is present in humans across the lifespan [14], and is also seen in primates [15], indicating evolutionary preservation and the involvement of genetic factors. In line with studies linking amygdala to the parcellation of stimulus’ relevance or significance [16], [17], a fast discrimination of novelty and a subsequent reallocation of sensory processing resources are essential to determine the significance of the event. However, few studies have investigated if the amygdala can influence the representation of novel events in visual cortex, indicating that amygdala’s ability to direct sensory and attentional resources goes beyond emotion to include a more general stimulus category. Further, most studies focusing on human amygdala and visual cortex responses to novelty have compared the activity to novel versus highly familiarized stimuli [7], [18], [19], though a recent study indicates that amygdala may be able to differentiate between a novel and a familiar stimulus already after one exposure [20], in line with electrophysiological recordings in the amygdala [6].

The amygdala is also a key structure in the detection of threat-related stimuli [21], [22], and may alter the selective attention to threats by influencing down-stream sensory networks. Interestingly, heightened state [23] anxiety is associated with an increased attentional bias toward threat-related stimuli, which has lead to the proposal that heightened state anxiety increases the output from threat detection networks [24], [25]. Though this association has been most studied for threat-related stimuli, some studies suggest that heightened anxiety may alter amygdala responses to other stimuli as well. For instance, subjects at risk of developing anxiety disorders have increased amygdala activity during evaluation of stimulus novelty [18], [26] or its approachability [27]. Essentially, it is possible that a more general category of behaviorally relevant stimuli provoke abnormal amygdala responses in anxious subjects, beyond emotional stimulation, subsequently affecting downstream brain areas and behavior.

The aim of the current study was to determine whether amygdala and the visual cortex differentiate levels of novelty. We investigated activity and functional connectivity between amygdala and extrastriate visual cortex during repeated presentations of an emotional face stimulus in subjects who underwent blood oxygen level-dependent (BOLD) functional magnetic resonance imaging (fMRI). Based on existing literature, we hypothesized that amygdala would differentiate novelty vs. familiarity already after a single stimulus exposure, while the visual cortex responses would have a more gradual signal decay. Further, we proposed that amygdala – visual cortex would be more functionally connected during 1^st^ time vs. 2^nd^ time stimulus presentation, reflecting modulation of visual cortex responses by the amygdala. Finally, individual variations in these pathways may be determined by state anxiety, ultimately explaining individual variations in physiological responses and behavior to stimulus novelty.

## Methods

### Subjects

The study was conducted at Oslo University Hospital, Norway, and approved by the Regional Committee for Medical Research Ethics and the Norwegian Data Inspectorate. Thirty-two subjects (14 women) aged 33.6±9.2 years participated in this study. The subjects were randomly selected from the Norwegian people registration (Statistics Norway) in the Oslo area and were invited by letter (32% response rate). All participants provided written, informed consent and received an honorarium. All subjects were screened with the Primary Care Evaluation of Mental Disorders (PRIME-MD), and excluded if they had a life time history of a psychiatric disorder or illicit drug abuse. Additional exclusion criteria included a medical condition known to interfere with brain function (i.e. hypothyroidism, uncontrolled hypertension and diabetes), neurological disorder or previous moderate to severe head trauma. Thirty-one of the participants completed the Spielberger State-Trait Anxiety Inventory (STAI) [28] immediately after the fMRI session. Because of the previously reported relationship between state anxiety and the amygdala-visual cortex pathways [24], [25], [29], only the state anxiety scores were used.

### Experimental Protocol

Twenty emotional faces from the NimStim series [30] depicting happy, angry, fearful or sad expressions were repeatedly presented during the experiment. In total, 55% of the faces expressed fear, 20% happiness, 10% anger and 15% sadness. Importantly, the percentage of each emotional subtype was balanced in our main contrast. The faces were of both genders (11 males). Each participant viewed a series of 69 sequentially presented faces for 2 s that were separated by a jittered inter-trial interval of 3.5±1 s in a randomised event-related design. The total number of presentations varied between 2–6 times for each face stimulus to avoid anticipatory responses in amygdala [31]. Further, direct repetition of the same face was avoided. Importantly, Balderston and colleagues found that amygdala activity diminishes already after a single presentation of an emotional stimulus, and that this effect remains consistent across subsequent trials [20]. Thus, we expected the signal drop in amygdala to be present also for stimuli that were only presented twice during the experiment. We employed an implicit gender decision task where subjects were instructed to give a right index finger response for every male face that appeared. The use of hands was counterbalanced. This task was chosen to ensure that subjects attended to the task, while at the same time avoiding cognitive challenges that would require regulation of amygdala dependent processes [32], [33].

We chose to study the effect of novelty by using the same picture in the novel and repeated conditions in line with other recent studies [20], [34]. This stands in contrast to earlier studies of amygdala and novelty, which often used different stimuli for the novel vs. highly familiarized conditions. However, without a condition where the images are repeated, it is more difficult to tell whether the effect is driven by novelty, or other qualities related to the stimuli belonging to the two different conditions. Thus we were able to control for any effects related to valence and stimulus identity. Though more novel stimuli were presented in the beginning of the experiment and the familiar towards the end, we sought to present novel stimuli throughout the whole time course. Further, we only used faces, as the novelty effect is consistently demonstrated for biological relevant stimuli [20]. Total scanning time was 388 seconds.

Following scanning, each subject rated all of the faces according to how intense they found each of the emotional expressions. The rating was performed on a laptop using a nine-point scale. Intensity was obtained due to its association with novelty [19] and amygdala activity [35], [36].

### Apparatus

E-prime software (Psychology Software Tools, Inc, Pittsburgh, PA) controlled the stimulus presentations with stimuli presented using VisualSystem (NordicNeuroLab, Bergen, Norway). Responses were collected using ResponseGrips (NordicNeuroLab, Bergen, Norway).

### Image Acquisition

MRI scans were acquired by a 1.5 T scanner (Siemens Magnetom Sonata, Siemens Medical Solutions, Erlangen, Germany) supplied with an eight channel head coil. In one session, 172 volumes (30 contiguous axial slices, each slice spanning 4 mm) covering the whole brain were acquired using an EPI BOLD sequence (TR = 2400; FOV 200×200 mm; 64×64 matrix; TE = 40 ms). In order to better localise our findings, T1-weighted anatomical images using an MPRAGE sequence (TR = 2000 ms; FOV 256×256 mm; 128×128 matrix; TE = 3.9 ms) were acquired.

### Behavioural Data Analyses

All the behavioural data was analysed in the Statistical Package for Social Sciences (SPSS 20.0. SPSS Inc., Chicago, USA). In order to compare if there were any differences in response times or accuracy for the 1^st^ time as compared to the 2^nd^ time presentation of the faces, paired-samples t-tests were performed. A possible association between the mean rated face intensity and the number of presentations for each face was tested using a Pearson product-moment correlation.

### Imaging Data

The images were visually inspected for signal dropout in the amygdala as this area is somewhat prone to magnetic susceptibility. However, none of our subjects had to be excluded due to signal dropout. The functional MRI data were pre-processed and analysed using the SPM8 software package (http://www.fil.ion.ucl.ac.uk/spm). All volumes were realigned to the first volume in the time series to correct for head motion [37]. One subject was excluded due to excessive head movements during the scan (cut off>3 mm). Subsequently, the mean functional image and the anatomical image were coregistered to ensure they were aligned. The images were spatially normalised to the Montreal Neurological Institute (MNI) template [38], resampled to 2×2×2 mm voxels and smoothed using a 6 mm full width-half maximum (FWHM) isotropic kernel. Data were high-pass filtered using a cut-off value of 128 s. To test for the effect of emotional novelty, we defined three event types; 1^st^ presentation (20 trials), 2^nd^ presentation (20 trials) and other presentations (consisting of the 3^rd^–6^th^ presentation trials, 29 trials in total). The model was specified by stick functions for the onsets of the three different event types, and convolved with a canonical hemodynamic response function. The contrasts of interest were “1^st^ presentation” > “2^nd^ presentation” and “2^nd^ presentation” > “other presentations”. For completeness, we also contrasted “1^st^ presentation” > “other presentations”. The individual contrast images were moved up to a second-level random effects model. Both the inferior occipital gyrus (IOG) and the fusiform gyrus (FFG) constitute key nodes in the face perception network [39], [40], and were therefore chosen as our *a priori* regions of interest in addition to the amygdala. Consequently, we used small volume correction (p_FWE_ = 0.05) based on anatomically defined (Automated anatomical labelling (aal) atlas in the SPM Wake Forest University (WFU) PickAtlas toolbox (http://fmri.wfubmc.edu/software/PickAtlas) [41]) bilateral IOG, FFG and the amygdala.

To identify regions where activity correlated with individual state anxiety scores, a second–level, linear regression model specifying the individual novelty responses (“1^st^ presentation” > “2^nd^ presentation”) and the log transformed STAI state anxiety scores as a covariate were used. Due to the consistent association between amygdala responsivity to threat-related stimuli and individual differences in state anxiety [24], [29], amygdala was our *a priori* region of interest. Thus we applied small volume correction (p_FWE_ = 0.05) based on anatomically defined (WFU Pickatlas; [41]) bilateral amygdala.

### Psychophysiological Interaction Analysis

To investigate if amygdala and visual cortex functional connectivity differed according to the novelty of the emotional faces, a psychophysiological interaction (PPI) analysis [42] was performed. It was hypothesized that amygdala and visual cortex would be more functionally connected during the 1^st^ time presentation of the faces relative to the 2^nd^ time presentation, based on their proposed roles in novelty processing. The current PPI had a design matrix incorporating a psychological variable (Novelty; 1^st^ vs. 2^nd^ presentation), the time-series of a seed region (right amygdala) and the interaction between the psychological and physiological variable. Only right amygdala was used as left amygdala displayed no significant responses in the second level analysis. For each subjecţ mean corrected activity was extracted from volumes of interest (first eigenvariate from a 6 mm sphere centred on the individual subject peak voxel within right amygdala. The individual peak voxels were localized within a 6 mm search region around the group peak voxel in right amygdala). The psychological variable represented the contrast between the 1^st^ and the 2^nd^ presentation states. The aim was to test for differences in regression slopes for two levels of novelty (i.e. 1^st^ and 2^nd^ presentation) as a measure of difference in regional connectivity (i.e. between seed region and other areas). To test for this, we generated a general linear model (GLM) in which the explanatory variable was the interaction term, and the main effects of time-course and the task regressors were included as covariates of no interest. The individual t-contrast images of the interaction gained from the PPI were then entered into a random effects one-sample t-test. IOG (BA 18) was defined as our region of interest in the visual cortex based on its suggested role as an entry node in the face-processing network [40], and thereof expected reactivity to faces novelty. Thus, small volume correction (p_FWE_<0.05) based on anatomically defined bilateral IOG (WFU Pickatlas [41]) was used to correct for multiple comparisons.

## Results

### Behavioural Results

Four subjects were excluded because their intensity-ratings were considered as outliers (>3SD) on more than three of the faces. The remaining twenty-seven subjects successfully completed the task (accuracy: 98.5±1.8%) and scanning procedure.

There was no significant difference in response time (t = 0.74, p = n.s.) between 1^st^ time and 2^nd^ time presentation of the faces. However, subjects performed significantly better during 2^nd^ time as compared to 1^st^ time presentation of the faces (t = −2.57, p = 0.02). Response times and accuracy by conditions are displayed in Table 1. In addition, no association between the mean intensity score of each face and the number of times the face had been repeated (r = −0.27, p = n.s.) was found.

**Table 1:** Accuracy and response time by conditions in the emotional faces task.

	Accuracy (%)	Reaction Time (ms)
1^st^ time presentation	94.3±0.9	638±26
2^nd^ time presentation	96±0.8	629±25

Participants’ state anxiety scores ranged from 20 to 48 (29.0±8.0). The state scores were not normally distributed and were therefore log transformed in SPSS before entering further analysis.

### Imaging Results

**Figure 1:**
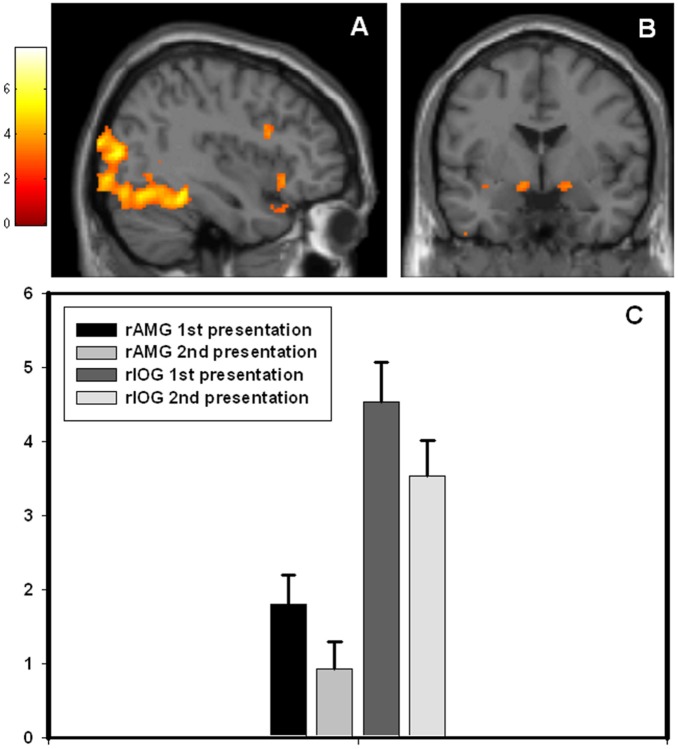
Amygdala and visual cortex BOLD activation to stimulus novelty. BOLD fMRI responses in the amygdala and visual cortex obtained for the contrast ”1^st^ presentation” >”2^nd^ presentation”. (A) Statistical parametric maps (SPM) demonstrating the responses in visual cortex for the given contrast. The image is thresholded at p = 0.005, k = 25 voxels for illustrative reasons. The colors refer to t-values as coded in the bar to the left of the image (B) Statistical parametric maps (SPM) demonstrating the responses in amygdala for the same contrast. The image is thresholded at p = 0.005, k = 25 voxels for illustrative reasons. (C) Beta values for the peak voxel in right amygdala (x = 24, y = −6, z = −14) and right inferior occipital gyrus (x = 34, y = −78, z = −12) for the conditions 1^st^ presentation and 2^nd^ presentation of the emotional faces.

**Figure 2:**
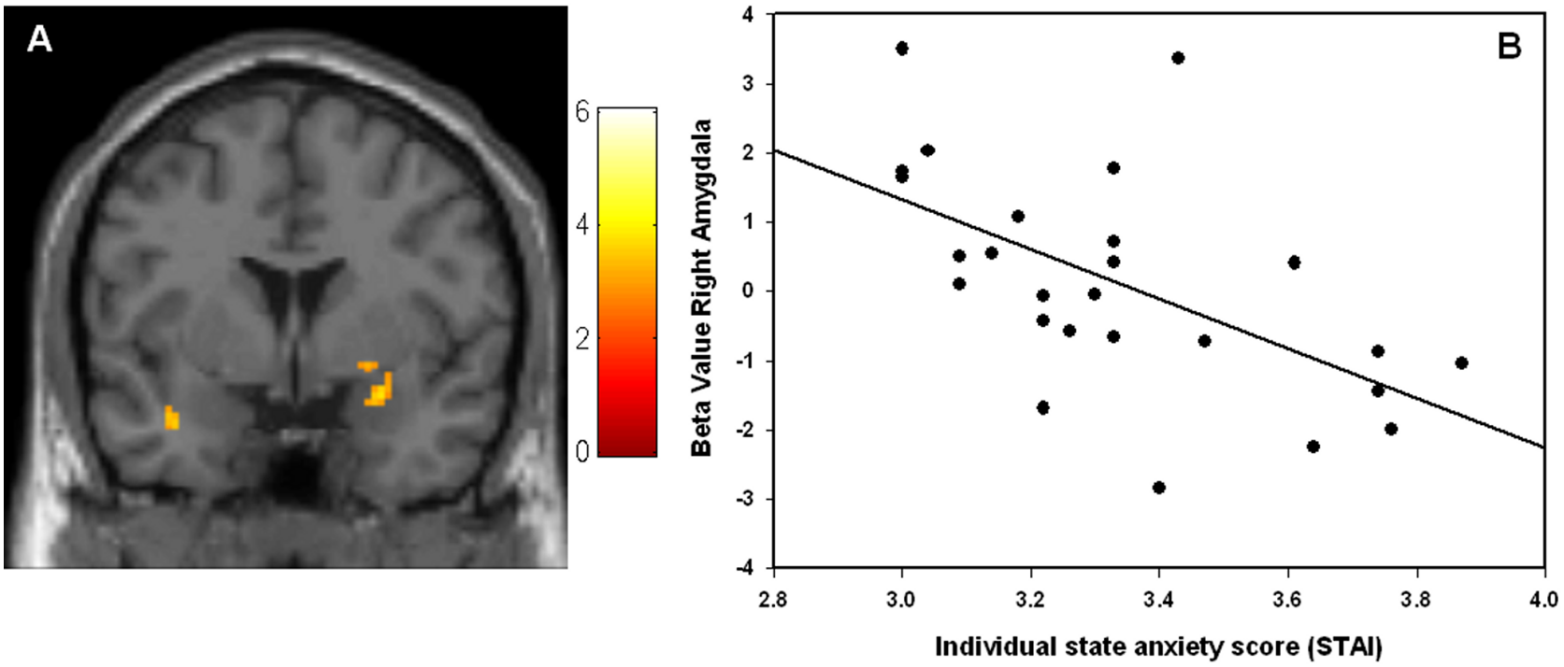
Correlation between state anxiety scores and individual amygdala activation. Negative correlation between the individual state anxiety scores and the activity in right amygdala. Subjects with a high score on state anxiety had less amygdala signal change between the 1^st^ time and the 2^nd^ time presentation of the emotional faces. (A) Statistical parametric map (SPM) showing the right amygdala cluster. The image is thresholded at p<0.005, 25 voxels extent threshold, for illustrative reasons. The colors refer to t-values as coded in the bar to the right of the image (B) Scatter-plot demonstrating the negative correlation.

Comparing 1^st^ vs. 2^nd^ time presentation of the stimuli, significantly increased activity in right amygdala and regions within the ventral visual stream including bilateral IOG and FFG were found for the 1^st^ time presentation (Table 2). The results are displayed in Figure 1. There was a significant negative correlation between right amygdala activity and individual state anxiety scores (peak voxel: x = 26, y = 2, z = −18, r = −0.55, p_SVC_ = 0.03, Figure 2), indicating that subjects with high scores on state anxiety had less signal change in amygdala from 1^st^ time to 2^nd^ time presentation of the faces. There was no significant association between left amygdala activity and individual state anxiety scores.

**Table 2:** BOLD fMRI responses in amygdala and visual cortex for the contrast “1^st^ presentation” > “2^nd^ presentation”.

	Hemisphere	Peak coordinates (MNI)	Peak Z	p_FWE_
Inferior Occipital Gyrus	Right	34, −78, −12	4.74	0.001
	Left	−44, −80, −6	5.56	<0.001
Fusiform Gyrus	Right	32, −78, −14	5.17	<0.001
	Left	−24, −76, −12	4.57	0.005
Amygdala	Right	24, −6, −14	3.16	0.02
	Left	−20, −1, −14	2.06	n.s.

Data are small volume corrected using anatomically defined bilateral amygdala, inferior occipital gyrus and fusiform gyrus (WFU Pickatlas [41]).

**Figure 3:**
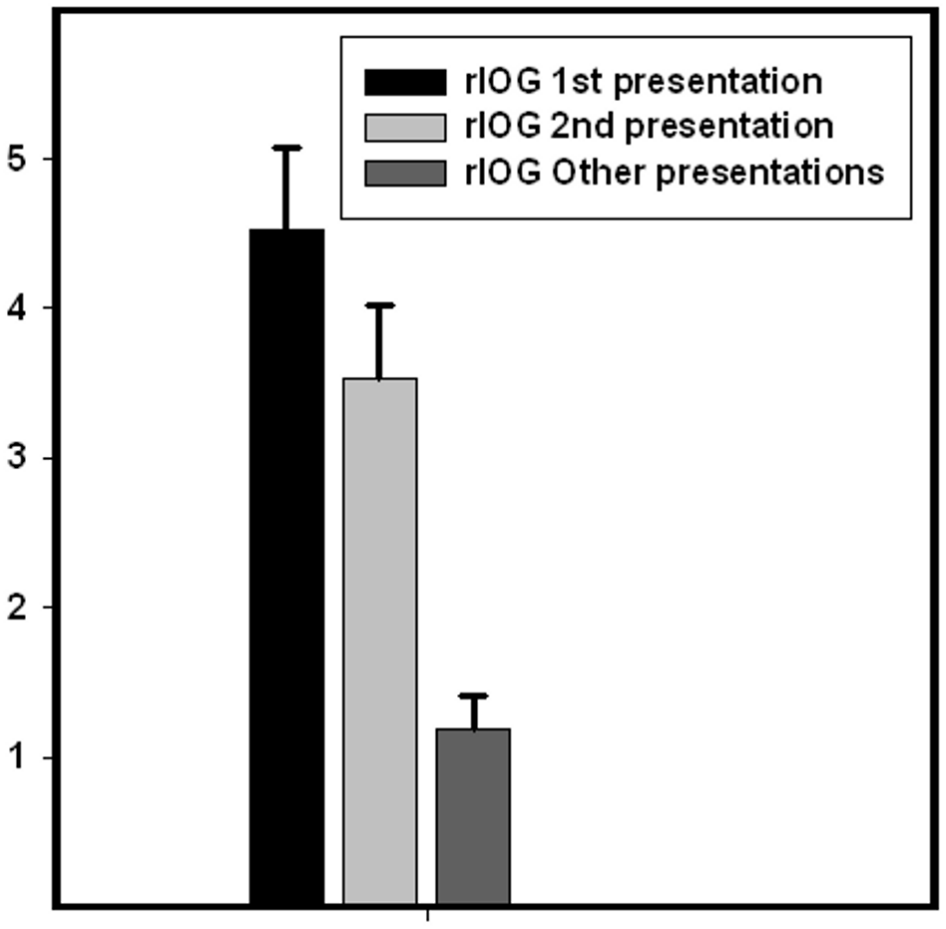
BOLD activation by condition in right inferior occipital gyrus. Beta values for the peak voxel in right inferior occipital gyrus (x = 34, y = −78, z = −12) for the conditions 1^st^ presentation, 2^nd^ presentation and other presentations of the emotional faces. The figure illustrates that the right inferior occipital gyrus BOLD fMRI response was significantly reduced in the 2^nd^ compared to the 1^st^ presentation. However, the BOLD response during the 2^nd^ presentation was significantly greater than the mean response from the remaining presentations of the faces.

We also compared responses for 2^nd^ time presentation and remaining presentations, and found significantly greater activity within the same regions of the visual cortex (right IOG peak voxel; x = 26, y = −92, z = −10, Z = 4.57, p_SVC_ = 0.002, left IOG peak voxel; x = −34, y = −86, z = −4, Z = 3.87, p_SVC_ = 0.02, right FFG peak voxel; x = 34, y = −48, z = −10, Z = 4.12, p_SVC_ = 0.03 and left FFG peak voxel; x = −40, y = −44, z = −24, Z = 4.20, p_SVC_ = 0.02) for the 2^nd^ time presentation. The responses in right IOG for the different conditions are displayed in Figure 3. However, there were no significant responses in the amygdala for this contrast, not even with a more lenient threshold (i.e. p = 0.05, uncorrected).

For completeness of data analysis, we also contrasted “1^st^ presentation” > “other presentations”. The results revealed significant increased responses in right amygdala (right amygdala peak voxel; x = 24, y = −8, z = −12, Z = 3.23, p_SVC_ = 0.02), bilateral IOG (right IOG peak voxel; x = 24, y = −92, z = −2, Z = 5.19, p_SVC_<0.001 and left IOG peak voxel; x = −48, y = −64, z = −16, Z = 4.84, p_SVC_<0.001) and bilateral FFG (right FFG peak voxel; x = 38, y = −42, z = −16, Z = 5.66, p_SVC_<0.001 and left FFG peak voxel; x = −22, y = −80, z = −12, Z = 5.39, p_SVC_<0.001) during 1^st^ time compared to other presentations, in line with the results from the main contrast. There was no significant responses in left amygdala for “1^st^ presentation” > “other presentations”.

### Psychophysiological Interaction Analysis (PPI)

**Figure 4:**
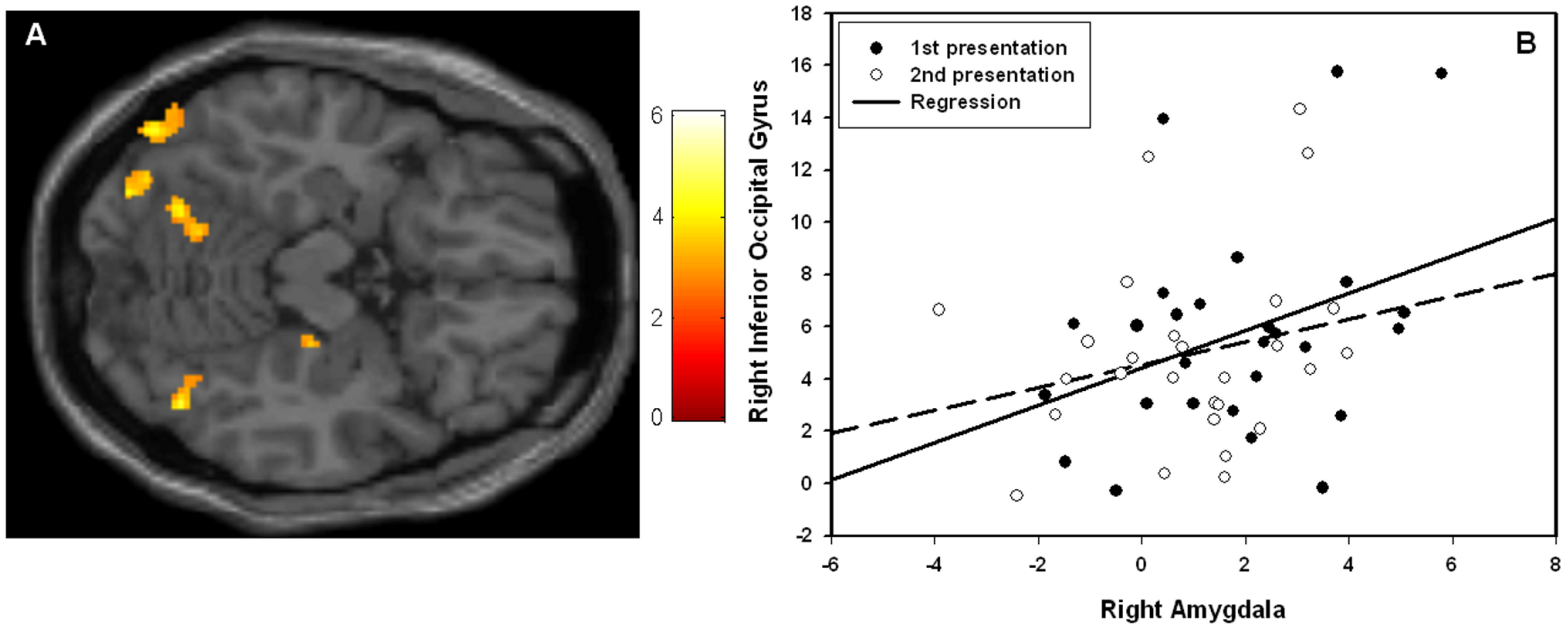
Psychophysiological interaction analysis. The results of the psychophysiological interaction analysis. (A) Statistical parametric map (SPM) showing regions in the visual cortex that showed condition-specific BOLD signal changes with right amygdala activity. The image is thresholded at p<0.005, 25 voxels extent threshold, for illustrative reasons. The colors refer to t-values as coded in the bar to the right of the image (B) Scatter plot with regression lines demonstrating the pattern of functional connectivity. The x-axis represents activity in the right amygdala (beta values) peak voxel and the y-axis represents activity in the right inferior occipital gyrus peak voxel (beta values).

The PPI-analysis with right amygdala as a seed revealed a significantly increased connectivity with right (peak voxel coordinate; x = 44, y = −66, z = −16, Z = 3.79, p_SVC_ = 0.03) and left (peak voxel coordinate; x = −28, y = −82, z = −12, Z = 4.04, p_SVC_ = 0.01) IOG during the 1^st^ as compared to the 2^nd^ time presentation of the emotional faces. The results are displayed in Figure 4.

## Discussion

In the present study, the effect of stimulus novelty on amygdala-visual cortex responses and connectivity were investigated. Enhanced responses were revealed in both amygdala and visual cortex for 1^st^ time presented compared to 2^nd^ time presented emotional faces. The effects in visual cortex were found all the way from extrastriate to occipitotemporal cortex, which collectively is referred to as the ventral visual stream. Interestingly, the amplitude of the amygdala response was associated with individual state anxiety scores, indicating that differences in these networks may exist based on state anxiety. Further, an increased functional connectivity between amygdala and IOG for the 1^st^ time presentation compared to 2^nd^ time presentation of emotional faces in a subsequent PPI analysis was obtained. The results support that the modulation of the visual system by amygdala goes beyond emotion to include novelty. Further, variations in these novelty detection pathways exist based on individual state anxiety, indicating that a person’s awareness and attention to novel events may rely on mood and personality traits.

The results from the second level analysis demonstrated increased BOLD-responses in large parts of the ventral visual stream, including bilateral IOG and FFG in addition to right amygdala, in response to 1^st^ time presentation of emotional faces. The increased responses in visual cortex may reflect amplified processing within sensory pathways mediated by the amygdala. Converging evidence from both animal and human research [12], [43] has highlighted such amygdala–sensory cortex projections as a source of top-down modulation of emotional perception based on their intimate structural [9], [10] and functional connectivity [44], [45]. Although most frequently studied in emotion, one study has reported that amygdala and visual cortex activity correlates during processing of novel stimuli [46]. Also, the amygdala BOLD response to novelty is often followed by an equivalent response in visual cortex [5], [20], indirectly supporting that the activity of these two brain areas covary during novelty detection. Taken together, the present results replicate previously reported relationships between activity in the amygdala and visual cortex in novelty detection, but extend these findings by demonstrating that amygdala-visual cortex functional connectivity varies already from 1^st^ to 2^nd^ time presentation of a stimulus. To the extent that these visual responses are directed by the amygdala, the current results support that amygdala’s ability to direct attentional resources extends to novel images, providing a neural substrate for the observed response patterns.

Interestingly, visual cortex activity also differentiated between 2^nd^ time presentation and subsequent presentations of a stimulus, while such activity was not found in the amygdala. This is in line with previous studies showing gradual signal decay in the inferior temporal cortex for repeated stimuli presentation [46]. Contrary, a recent study by Baldenston and colleagues reported that activity in amygdala diminished already after a single stimulus exposure, and that this difference remained consistent throughout subsequent trials [20]. The more gradual decline of visual cortex responses may be due to modulation by prefrontal cortices that selectively amplifies visual cortex responses to attended stimuli at the expense of other representations [47]. Alternatively, it is possible that an initial significance labelling provided by the amygdala primes neurons in the visual cortex [24]. Consequently, with repeated exposure, the visual cortex may continue firing above baseline until a certain point in time when the stimulus significance declines.

In the present study, activity in right amygdala covaried more strongly with bilateral IOG when processing novel as compared to familiar faces. The finding is supported by other functional connectivity analyses demonstrating covariation between activity in the visual cortex and amygdala as a function of emotional awareness [44], valence [45] and attentional set [48]. However, the effect of novelty on this functional connectivity has, to our knowledge, not been previously examined. There are at least two possible interpretations of this finding. One interpretation is that amygdala both directly and indirectly, via frontoparietal regions, modulates responsiveness in IOG [11]. Alternatively, another independent set of brain areas code novelty, and further modulates the amygdala-visual cortex projections accordingly. Novelty detection is supported by a network of brain regions including the medial temporal lobe, visual, parietal and prefrontal cortices in addition to the dopamine midbrain [4], [49]. Both amygdala and visual cortex are tightly interconnected with parts of the prefrontal cortex and dopamine midbrain [50], [51], thus making these two areas candidate regions for mediating this effect. Although the current data doesn’t allow us to exclude this last interpretation, the literature has suggested that the amygdala plays an important role in the neural circuitry coding for novelty [5], [6], [7], [8]. Further, amygdala has direct projections to all parts of the ventral visual stream [9], [10] and is known to modulate neuronal activity in these brain areas based on stimulus emotional properties [52]. When considering these different functions of the amygdala together, it is possible that excitatory feedback from the amygdala in response to novel emotional stimuli during task performance could cause the observed enhanced connectivity between the amygdala and visual cortex.

Subjects performed significantly better during 2^nd^ time presentation compared to 1^st^ time presentation of the faces without any changes in response times. This is in keeping with studies reporting that animal behavior can be modified by a single exposure to a relevant stimulus [53]. Findings from electrophysiological studies in humans have elaborated this finding by showing that neurons in the amygdala and hippocampal complex obtain information sufficient to distinguish novel from familiar stimuli already after a single exposure, and these neurons retain their memory for 24 hr [6]. Thus it is possible that recognition memory for the 2^nd^ time presented faces, which is a highly automatic form of memory, contribute to the observed behavioral improvement.

Previous studies have demonstrated that elevated state anxiety is associated with increased amygdala responsiveness to unattended threat-related stimuli [29], especially under low perceptual load [25]. Further, greater [18], [54] and sustained [26] amygdala responsiveness to novelty has been related to inhibited temperament, which is a risk factor for developing anxiety in both childhood [55] and adolescence [56]. The present study adds to this understanding by demonstrating that not only amygdala’s threat-related responses vary according to measures of anxiety, but more generally the amygdala’s novelty-related response. Subjects with high scores on state anxiety demonstrated less signal difference between 1^st^ and 2^nd^ time presentation of the emotional faces, in line with findings demonstrating less habituation of amygdala responses in subjects with a high score on temperamental inhibition [26]. Thus, if the amygdala updates the relevance or significance of a stimulus during familiarization, this process may be abnormal or less efficient among highly anxious subjects.

Related to novelty responses is the habituation effect often observed in the amygdala to emotional stimuli [57], [58]. Based on the temporal dynamics of the habituation response, we do not think the present results reflect amygdala habituation effects. Generally, amygdala habituation implies more gradual signal decay towards a baseline, opposite to the almost immediate return, as expected for novelty specific responses [20], [59]. As we did not find any significant amygdala responses when comparing the 2^nd^ time presentation with the remaining presentations of the faces stimuli, it supports novelty related activity in the amygdala. In line with this, novelty is suggested as a critical stimulus dimension for amygdala engagement, independent and additive to emotional values [19], [20], [34].

A strength of the current study was that the stimuli belonging to the two conditions did not differ in visual complexity, valence or identity. This may be a confounding factor when studying possible top-down modulation of visual cortex by the amygdala, as the observed effects might be due to different processing demands on visual cortex or emotion encoding in the amygdala. It has been suggested that novel stimuli are more arousing than familiar ones [19]. However, rating of emotional intensity did not differ based on how often the visual stimulus had been presented during the task. Notably, the present study design does not allow us to fully exclude adaptation effects in the amygdala. To prevent stimulus adaptation, we avoided direct repetition of a specific face. Still, adaptation to repeated presentation of one emotional subtype, especially fear, could potentially be present. Arguing against this, however, is the observation that fear adaptation in the amygdala may evolve across several trials, and potentially only for the behavioural relevant stimuli [60]. Furthermore, novelty responses in the amygdala have been consistently observed for emotional neutral stimuli as well [5], [7], [34], and a recent study failed to find additive effects of emotion to these novelty specific responses in the amygdala [34]. Most studies of emotional novelty compare amygdala activity for novel and highly familiarized stimuli using block-design experiments, in which responses are averaged across several stimulus repetitions in the familiarized condition. However, if the responses are driven by novelty, then the magnitude of the response should not depend on number of times the stimuli are being repeated [20]. Hence, our finding that amygdala and visual cortex may differentiate between novel and familiar events already after one presentation supports that these brain regions constitute a sensitive and interacting novelty detection network.

### Conclusion

In summary, the present study demonstrates that amygdala and visual cortex are able to differentiate the novelty of emotional stimuli already after one presentation. Further, variation in functional connectivity between these areas for the same contrast indicates that their interactions are crucial for rapid and sensitive discrimination of stimulus’ novelty. The amygdala response varied based on individual differences in state anxiety, supporting that variation in these networks as a vulnerability factor for psychopathology goes beyond emotions to include a broader category of environmental stimuli.
